# Breast Cancer and the Radiation Oncologist

**Published:** 1989-05

**Authors:** Sally Goodman

**Affiliations:** Department of Radiotherapy, Bristol Royal Infirmary


### Breast Cancer and the Radiation Oncologist: A Personal Comment

**Sally Goodman M.A., M.R.C.P., F.R.C.R.**
Department of Radiotherapy, Bristol Royal Infirmary

Women with breast cancer make up 20 to 25% of new referrals to the Bristol Radiotherapy and Oncology Centre each year. Most come as secondary referrals from surgeons but a small number, often elderly and infirm, or with widespread metastatic disease, will come from physicians or general practitioners. Many patients with breast cancer will ultimately die of their disease, but the time course may be protracted, with a significant number relapsing more than 10 years after diagnosis. This means that patients will be under our care for many years: undergoing primary treatment, coming for follow up, having further radiotherapy or other treatment for their metastatic disease, and finally, coming to us for terminal care if other options are not available.

Breast cancer is not a single disease process, and it can produce many surprises. Disease tempo covers a wide range, and it can be difficult to determine whether a particular treatment is having an impact on survival or simply altering the time course of different phases of the disease. Adjuvant treatment can delay the time to development of the first metastasis but subsequent treatment is often less effective, and overall survival unaffected. Before we can decide whether new approaches to treatment offer any advantages, we need to look at the results of trials involving large numbers of patients followed up for many years.

### EARLY DISEASE

The primary approach to the treatment of breast cancer is surgical. Mastectomy, simple or radical, is carried out less often now, but it still has its place. However, local excision of the primary tumour followed by radiotherapy seems to be equally effective in providing local control where the tumour is fairly small, well circumscribed and not fixed to other structures. The extent of local excision varies enormously, but the risk of local recurrence is considerably increased if there is residual tumour after any procedure. Great care must be taken over the shape and siting of the scar if the best cosmetic results are to be achieved (and this surely is the purpose of breast preservation). Radiotherapy cannot improve cosmesis, and may make it worse. An irregular, scarred breast is not only unsightly, but may be uncomfortable or painful, producing fears of recurrent disease and making follow-up assessment difficult.

After surgery there are two factors to consider: reduction of the risk of local recurrence, and reduction of the risk of developing metastases.

In spite of considerable difficulties in confirming results with adequate randomised trials it seems safe to say that the approach to local disease, so long as it provides adequate local control, has no effect on survival. At present this means mastectomy, with local radiotherapy only if there are poor prognostic factors for recurrence, or local excision followed by radiotherapy in all cases. There are no groups so far identified in whom radiotherapy after local excision can be omitted. Local recurrence has been seen in patients presenting with tiny tumours or even with carcinoma in situ. Often the state of the axillary lymph nodes is unknown after lumpectomy so a useful prognostic indicator is missing.

Radiotherapy starts 3 to 4 weeks after surgery if possible, to allow healing to take place, bruising to settle and shoulder mobility to improve. In Bristol patients usually receive 4 to 5 weeks treatment to the breast and lymph nodes. Other centres may use longer or shorter courses of treatment, which are, however, biologically equivalent. The length of the course is partly dictated by the constraints of machine time and staffing but, in general, longer courses produce less long term damage to normal tissue. Treatment is completed with a boost dose to the tumour bed. This can be given by external irradiation, or by a temporary implant of radioactive wire (iridium-193).

Radiotherapy is usually well tolerated. Patients will feel tired, and sometimes nauseated or depressed. Many are able to drive themselves in for treatment and continue with a modified version of their daily routine, be it at home or in employment. The skin reaction, which develops towards the end of treatment and reaches its peak about a week after completion, is rarely severe with mega-voltage equipment.

The prevention of metastatic disease is still a contentious issue. Over the past 30 years there have been numerous trials of adjuvant treatment with hormone manipulation or chemotherapy. Although the time to relapse may be extended, few have shown any survival advantage. Analysis of the pooled results of a number of trials suggests that the benefits of treatment may be greater than would appear from individual trials, but we still do not know which is the best treatment, who will benefit most, and what the long term risks of such treatments would be if they were to be given to large numbers of women. Many women are given adjuvant hormone therapy because it is simple to take and relatively free of side effects. Some of these women might do better with chemotherapy, but the increased complexity of treatment and the often unpleasant side effects make us consider this decision much more carefully. In practice, adjuvant chemotherapy is usually reserved for patients with very aggressive tumours. This group is easy to identify, but will not necessarily get the most benefit. Current research is being directed towards the identification of sub-groups with more subtle characteristics that may auger a worse prognosis.

### LATE DISEASE

Treatment of late disease is less contentious because the patient is now incurable. The aim should be to palliate symptoms in a way compatible with achieving the best quality of life. Having said this, patients who relapse late, with limited local disease or metastases in bone, are more likely to respond to hormone manipulation, and may survive many years. Those who relapse early in multiple visceral sites (especially brain or liver), and who are pre or perimenopausal, are much less likely to respond to hormones and can be expected to have a median survival of less than 2 years. Chemotherapy can be useful in palliation in this group and it is possible to choose single drugs or less aggressive combinations with acceptable side effects.

Radiotherapy is the mainstay of treatment for bony metastases, and can often control symptoms from brain, lung and nodal disease. When giving palliative treatment, courses lasting one to ten days are the rule.

Palliative treatment should be tailored to the individual and her problems. Wherever possible she should take part in decisions about treatment.

### GENERAL POINTS

There has been considerable publicity about all aspects of breast cancer, some of it undoubtedly distressing to patients. Equally, much of it has helped to improve communication.

